# Practical applications of methods to incorporate patient preferences into medical decision models: a scoping review

**DOI:** 10.1186/s12911-025-02945-5

**Published:** 2025-03-03

**Authors:** Jakub Fusiak, Kousha Sarpari, Inger Ma, Ulrich Mansmann, Verena S. Hoffmann

**Affiliations:** The Institute for Medical Information Processing, Biometry, and Epidemiology, Ludwig-Maximilians-Universität, Marchioninistr. 15, 81377 Munich, Bavaria Germany

**Keywords:** Decision-making, Key concepts, Medical decision models, Patient preferences, Scoping review

## Abstract

**Background:**

Algorithms and models increasingly support clinical and shared decision-making. However, they may be limited in effectiveness, accuracy, acceptance, and comprehensibility if they fail to consider patient preferences. Addressing this gap requires exploring methods to integrate patient preferences into model-based clinical decision-making.

**Objectives:**

This scoping review aimed to identify and map applications of computational methods for incorporating patient preferences into individualized medical decision models and to report on the types of models where these methods are applied.

**Inclusion Criteria:**

This review includes articles without restriction on publication date or language, focusing on practical applications. It examines the integration of patient preferences in models for individualized clinical decision-making, drawing on diverse sources, including both white and gray literature, for comprehensive insights.

**Methods:**

Following the Joanna Briggs Institute (JBI) methodology, a comprehensive search was conducted across databases such as PubMed, Web of Science, ACM Digital Library, IEEE Xplore, Cochrane Library, OpenGrey, National Technical Reports Library, and the first 20 pages of Google Scholar. Keywords related to patient preferences, medical models, decision-making, and software tools guided the search strategy. Data extraction and analysis followed the JBI framework, with an explorative analysis.

**Results:**

From 7074 identified and 7023 screened articles, 45 publications on specific applications were reviewed, revealing significant heterogeneity in incorporating patient preferences into decision-making tools. Clinical applications primarily target neoplasms and circulatory diseases, using methods like Multi-Criteria Decision Analysis (MCDA) and statistical models, often combining approaches. Studies show that incorporating patient preferences can significantly impact treatment decisions, underscoring the need for shared and personalized decision-making.

**Conclusion:**

This scoping review highlights a wide range of approaches for integrating patient preferences into medical decision models, underscoring a critical gap in the use of cohesive frameworks that could enhance consistency and clinician acceptance. While the flexibility of current methods supports tailored applications, the limited use of existing frameworks constrains their potential. This gap, coupled with minimal focus on clinician and patient engagement, hinders the real-world utility of these tools. Future research should prioritize co-design with clinicians, real-world testing, and impact evaluation to close this gap and improve patient-centered care.

**Supplementary Information:**

The online version contains supplementary material available at 10.1186/s12911-025-02945-5.

## Introduction

Patient-centred care is supposed to foster a partnership between patients and healthcare providers (e.g. nurse practitioners, physician assistants, nurse anesthesiologists) [1]. However, patient autonomy can still be undermined when patient preferences do not align with treatment options or outcomes. Studies have shown that this misalignment can lead to lower patient satisfaction [2], reduced treatment adherence [3], and poorer health outcomes [4]. While some patients prefer active participation in treatment discussions, others may prefer to rely entirely on their physicians for decision-making [5].

Shared decision-making (SDM) is a model designed to enhance patient autonomy by combining the medical expertise of healthcare professionals with the personal preferences of patients to make informed treatment decisions [6]. Despite its benefits, implementing SDM can be challenging due to practical barriers such as time constraints and a lack of training for healthcare providers on how to effectively engage with patients [7]. Medical decision models can help to overcome these barriers by directly integrating patient preferences paving a structured way to SDM for patients and doctors alike [8]. These models are systematic frameworks or tools designed to support clinical decision-making by incorporating medical evidence, clinical expertise, explicit patient preferences, and individual patient data, such as laboratory results or disease stage.

Examples for medical decision models are clinical decision aids and support systems, at multiple stages of the decision process including patient-clinician discussions, preference-based treatment selection, and final implementation [9]. Ideally, this approach should provide patients with active involvement in the decision-making process, clear information on treatment side effects, needed adherence, and possible outcomes, and promote discussions with the attending healthcare provider [10]. Additionally, these tools can help save costs [11], limit litigation [12], and reduce health inequality [13].

Although there are studies that have already reviewed certain aspects of incorporating patient preferences for medical decision-making [9, 14, 15], our approach offers a comprehensive overview of already existing methods and identifies current trends of certain methods and gaps where further research or a paradigm change may be needed to enhance patient-centered care and SDM. We want to highlight the use of medical decision models that align medical decisions with patient values and preferences by giving two examples:

The first example is a model for patients with severe stroke to aid the decision on whether to perform a decompressive craniectomy including patient preferences. Lazaridis and Mansour [16] developed a decision-making model based on Expected Utility Maximization (EU). The authors addressed the need for an individualized approach by presenting scenarios in which patients expressed diverse preferences for post-treatment outcomes, such as the degree of acceptable disability or the emphasis on survival. EU was applied to rank potential outcomes according to each patient’s specific values, rather than standard clinical metrics. This framework underscores the importance of accommodating personal preferences in medical decisions to achieve outcomes that patients find meaningful and acceptable.

The second example, a tool for multiple sclerosis (MS) patients developed by Kremer et al. [17]. The tool uses Multi-Criteria Decision Analysis (MCDA) to support patients in choosing among disease-modifying drugs (DMDs). It integrates patient preferences across multiple treatment attributes, including efficacy, safety, and ease of use. By applying MCDA, Kremer et al. enabled patients to weigh these treatment characteristics according to their own priorities, enhancing shared decision-making. Early testing showed that patients felt more informed and engaged in their treatment choices, illustrating the practical utility of MCDA in facilitating preference-sensitive decisions.

In this scoping review we have investigated medical decision models for clincial specific applications which are based on computational methods and incorporate patient preferences. Computational methods are defined as algorithmic processes that are used to model decision-making based on quantitative data, including methods such as statistical models, decision analysis tools, or artificial intelligence approaches.

## Methods

### Protocols and registration

The study followed the Joanna Briggs methodology. The study was registered at Open Science Framework (https://osf.io/qg3b5). The proposed scoping review was conducted in accordance with the JBI methodology for scoping reviews and in line with the Preferred Reporting Items for Systematic Reviews and Meta-Analyses extension for Scoping Reviews (PRISMA-ScR); see Appendix B. A review protocol is available at the JBI Evidence Synthesis database describing the methodology of this study in detail [18]. The inclusion and exclusion criteria for this review were predefined in this protocol, which provides a detailed account of the methodology, including criteria applied during the screening process. It outlines all criteria used to determine study eligibility to ensure a transparent and replicable screening process.

### Inclusion criteria

This scoping review included studies involving any type of patient and focused on integrating patient preferences into medical decision-making algorithms and models. The concept encompasses methods for quantifying, balancing, and evaluating patient preferences using structured models or step-by-step algorithms to support clinical decisions. These methods include techniques designed to incorporate patient preferences into personalized treatment recommendations. Additionally, the review considered evidence addressing the limitations of these methods and their future research needs, as described in the literature. The context of this review was decision-making in clinical healthcare settings, with an emphasis on computational algorithms and models tailored to individual patient preferences. There were no geographical restrictions, and the focus was on enhancing personalized medicine by aligning treatment decisions with patient-specific goals and preferences. A wide range of sources was considered, including peer-reviewed journal articles and gray literature, such as original research, qualitative and quantitative studies, mixed methods studies, conference abstracts, dissertations, theses, technical reports, clinical practice guidelines, policy documents, and expert opinions. This diverse range of sources provided a comprehensive overview of the evidence related to the integration of patient preferences into computational decision-making models. This scoping review included all relevant articles describing computational methods for incorporating patient preferences into personalized treatment recommendations in clinical environments based on a model. Eligible sources included original research articles, reviews, conference abstracts, dissertations, theses, technical reports, clinical guidelines, policy documents, online resources, and expert opinions obtained through personal communication. Systematic reviews were excluded; however, references from these reviews and included articles were screened for additional evidence. There were no limitations regarding the year of publication to ensure a comprehensive overview of the field. No restrictions were placed on the language of publications. Translation software was employed to facilitate comprehension of non-English articles. Articles that could not be accurately translated using this method were excluded, though this was not necessary as all retrieved articles were in English. Authors of missing or not accessible articles were contacted up to three times via email or the social media application ResearchGate to request additional or missing data.

### Literature search

A literature search was performed by JF and coauthors in September 2023 in multiple databases, including PubMed, Web of Science, ACM Digital Library, IEEE Xplore, the Cochrane Library, for white publications. OpenGrey, the National Technical Reports Library, and the first 20 pages of Google Scholar, sorted by relevance, were used for feasibility reasons to find gray publications. Some data-base-specific adaptations were made to the search strategy for the different databases. Detailed information on the literature search is provided in Appendix C.

### Screening, data charting process, and synthesis of results

After a pilot test of 10 publications to ensure data consistency between reviews and refinement of the data extraction tool titles and abstracts were screened by at least two independent reviewers to assess inclusion criteria. The criteria and data of interest were defined using qualitative content analysis as per Mayring [19], using publications from the pilot test. All identified citations were uploaded to Rayyan (Qatar Computing Research Institute, Doha, Qatar; [20]) for blind screening and duplicate removal. Relevant sources were recovered and the findings were imported into the data extraction tool. REDCap (Vanderbilt University; [21]) was used as the data extraction tool to ensure data consistency and safety. Full-texts of selected citations were independently evaluated on the basis of inclusion criteria, with reasons for exclusion documented. Disagreements were resolved by discussion between authors J.F, K.S and I.M. The data extracted covers publication year, origin country, incorporated patient preferences, definitions of patient prefernces, methods for assessment and incorporation of patient preferences, methods for evaluation, balancing, and quantifying patient preferences, outcomes, benefits, limitations, and implications for further research. In the preparation of this manuscript, ChatGPT version 4o, a Large Language Model (LLM) developed by OpenAI, was used to enhance the language and readability of the text.

## Results

### Study inclusion

**Fig. 1:**
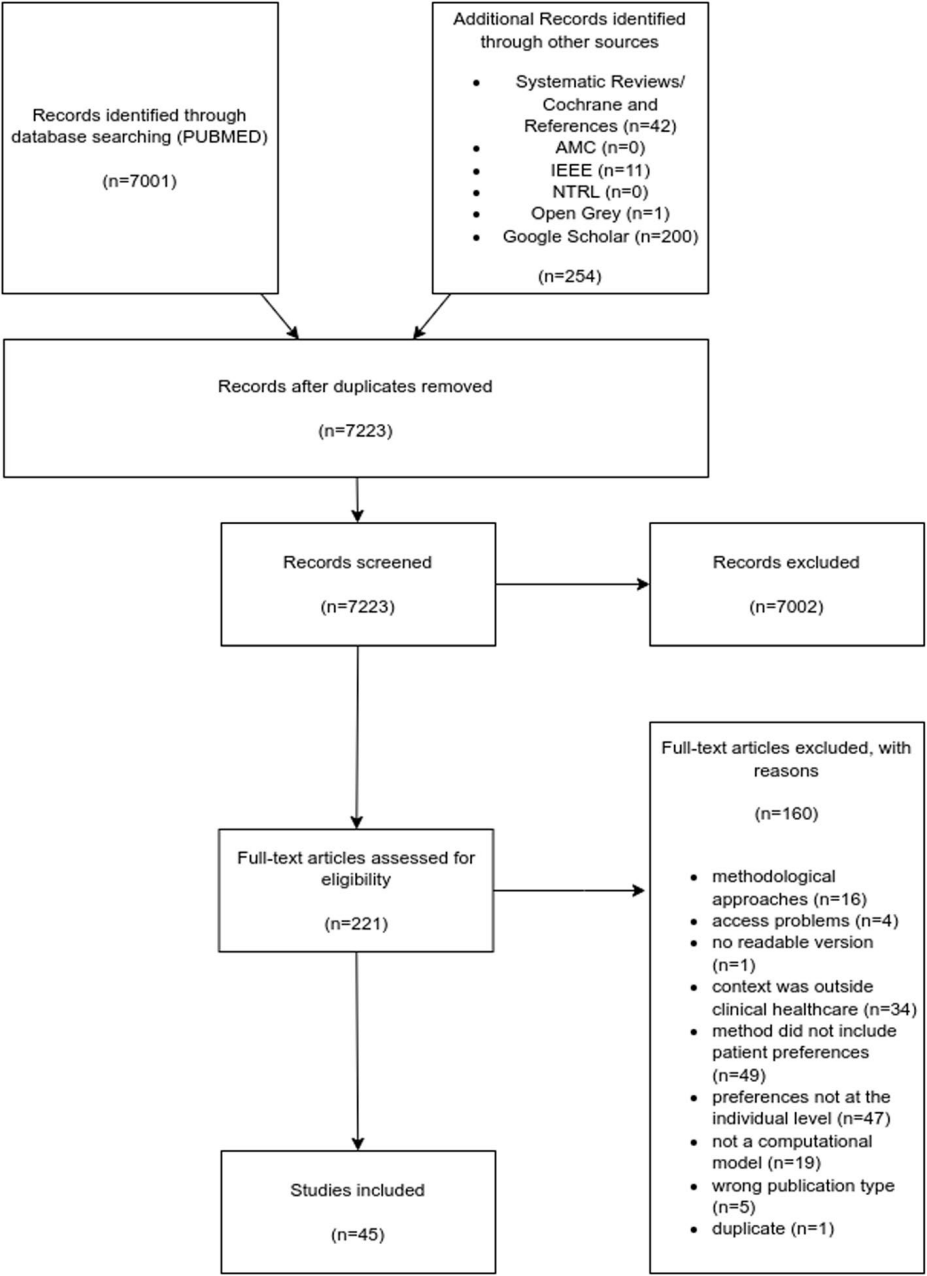
PRISMA flow chart illustrating process of the scoping review

Details related to search results and source selection in this review can be found in the PRISMA flow diagram in Fig. 1. The searches yielded 7255 records. After duplicates were removed, 7223 titles and abstracts were screened. This included 200 results from the first 20 pages of Google Scholar, which were screened manually within Google Scholar due to the lack of an export function to Rayyan. In total, 7002 records were excluded since they were not related to the research question. A total of 23 references from systematic reviews were screened. While none of the systematic reviews directly addressed our research question, a few were tangentially related to our investigation. From their references, we identified 7 relevant studies, which were included in the final analysis to ensure a comprehensive mapping of computational methods for incorporating patient preferences. We selected 221 full-text reports and from these, 45 met the inclusion criteria and were included in the study. This review is focused only on practical clinical applications as we aimed to investigate methods which are currently used for decision-making in practice. Data from the 16 methodological articles containing methodological approaches were therefore excluded. Appendix D presents data on specific applications.

Five publications could not be reviewed due to access problems to the publication or inability to find a readable version and thus were excluded. Additionally, as detailed in the PRISMA flowchart (Fig. 1), we excluded studies that were methodological in nature, of an unsuitable publication type (e.g., scoping reviews, books), focused on contexts outside clinical healthcare, or employed methods that did not incorporate patient preferences or did so only at the population level.

### Characteristics of included sources

**Fig. 2:**
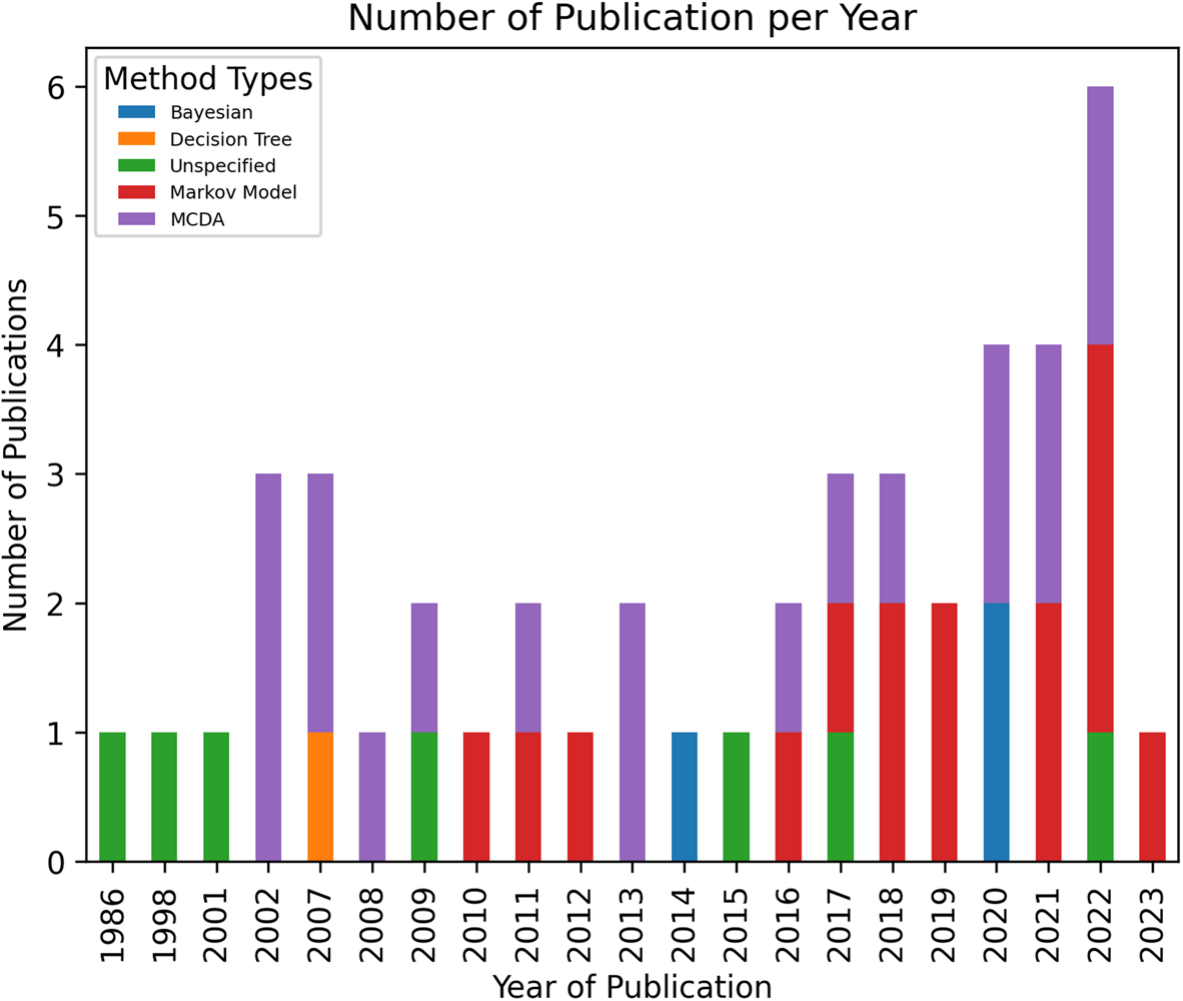
Stacked bar chart showing the number of studies per year, categorized by different methods. The x-axis represents publication years, and the y-axis shows the number of publications, with each color indicating a specific method

Of the 45 references included, all reports were published in English and the first authors were mainly affiliated with the United States (n = 27; 44.27%), UK, (n=8; 13.11%), Canada (n=6; 9.84%), and Australia (n=4; 6.56%). Our analysis (Fig. 2) shows that this topic has gained traction in recent years, especially since 2017, as nearly half of the methods have been published during the last eight years. A complete list of the characteristics of included sources can be found in Appendix D.

### Definition of patient preferences

Patient preferences have evolved significantly in healthcare literature from 1986 to 2023, reflecting shifts in conceptualization and an increasing emphasis on personalization. Definitions of patient preferences can be grouped into the following categories:

- **Utility-Based Definitions:** Patient preferences are defined as numerical representations of the value assigned to different health outcomes. These definitions emphasize the importance of incorporating individual preferences into decision-making processes to reflect patient-specific priorities and trade-offs between health states.
  - *Example:* A patient with chronic pain might assign a utility score of 0.8 to a treatment that alleviates pain but causes mild nausea and a score of 0.5 to one that eliminates pain but results in severe fatigue. This approach illustrates the quantification of patient preferences for different health outcomes [16, 22–29].
- **Trade-Off Focused Definitions:** These definitions highlight how patients evaluate competing outcomes by balancing risks, benefits, and burdens. They emphasize the subjective nature of preferences and the prioritization of certain outcomes over others based on individual goals and values.
  - *Example:* A cancer patient may prioritize a treatment with a 70% chance of remission but a risk of severe side effects over one with a 50% chance of remission and minimal side effects. This reflects the trade-offs patients are willing to make between potential outcomes [30–36].
- **Elicitation-Based Definitions:** These definitions focus on the process of systematically capturing preferences as expressions of values and priorities. They emphasize the act of eliciting what is most important to the individual, creating structured representations of their priorities for decision-making. This category views preferences as inputs derived from focused elicitation efforts.
  - *Example:* Using a discrete choice experiment, a patient with diabetes selects between treatment options varying in administration frequency, effectiveness, and potential side effects to express their preferences. This captures the patient’s values in a structured manner [17, 37–40].
- **Context-Specific Definitions:** These definitions acknowledge that preferences are not static but vary based on the clinical context, condition, and individual circumstances. They underscore the need for tailored approaches to understanding preferences.
  - *Example:* A stroke survivor might prioritize regaining mobility over reducing the risk of future strokes, while another may prioritize minimizing medication burden. This variation demonstrates how preferences are shaped by specific contexts [41–43].
- **Value Alignment Definitions:** Patient preferences are conceptualized as the alignment of healthcare decisions with individual values, goals, and priorities. This category extends beyond capturing preferences to ensuring that decisions are consistent with what matters most to the patient. These definitions emphasize the integration of preferences into broader ethical and personal contexts, serving as the foundation for shared decision-making and patient-centered care.
  - *Example:* A patient nearing the end of life might prioritize comfort and spending time with family over pursuing aggressive treatments with uncertain benefits. This illustrates the alignment of medical decisions with deeply held personal values [44–48].

### Use cases

This review will focus on specific applications [16, 17, 22–64], as they provide deeper insight into the clinical implementations of different methods and their interaction with clinicians, patients, and other elements of the clinical infrastructure.

We assigned an ICD-11 category (International Statistical Classification of Diseases and Related Health Problems) to each publication to investigate which medical conditions already have existing methods. Table 1 shows that most applications have been developed for neoplasms (e.g. prostate cancer (n=4; [24, 34, 37, 51]), breast cancer (n=2; [26, 32], or ovarian cancer (n=2; [22, 49]), diseases of the circulatory system (e.g. atrial fibrillation (n=4; [33, 35, 43, 58]), or chronic limb-threatening ischemia (n=2; [48, 62]), and diseases of the nervous system (e.g. multiple sclerosis (n=2; [28, 61]).

**Table 1:** ICD-11 categorization of medical conditions of found specific applications

ICD-11	Description	Number of findings	References
2	Neoplasms	n=9	[24, 26, 30, 32, 34, 37, 49, 51, 56]
11	Diseases of the circulatory system	n=9	[23, 33, 35, 43, 44, 48, 58, 61, 62]
8	Diseases of the nervous system	n=6	[16, 17, 25, 28, 29, 42]
16	Diseases of the genitourinary system	n=3	[30, 41, 63]
6	Mental, behavioural or neurodevelopmental disorders	n=3	[38, 52, 57]
15	Diseases of the musculoskeletal system or connective tissue	n=3	[45, 47, 53]
24	Factors influencing health status or contact with health services	n=3	[31, 36, 60]
5	Endocrine, nutritional or metabolic diseases	n=3	[46, 55, 59]
3	Diseases of the blood or blood-forming organs	n=2	[40, 54]
18	Pregnancy, childbirth or the puerperium	n=1	[64]
22	Injury, poisoning or certain other consequences of external causes	n=1	[27]
21	Symptoms, signs or clinical findings, not elsewhere classified	n=1	[39]

### Type of decision support

Our review identified a wide range of decision-making types that incorporate patient preferences: models (n=19; [16, 22–28, 30, 31, 34, 44, 48, 49, 52, 55, 56, 60–62]), decision aids (n=20; [17, 32, 33, 35, 35, 37, 39–41, 43, 45–47, 49–51, 53, 63, 64]), and decision support systems (DSS) (n=6; [29, 39, 45, 46, 54, 56, 57, 59]). **Models** predict various outcomes based on different clinical inputs and patient preferences, thereby aiding in more accurate and personalized medical planning. Our findings show that mostly various decision analysis models are used to incorporate patient preferences for decision-making. While some models may be interactive, their primary goal is computation and prediction. **Decision aids** are interactive tools designed to help patients understand their medical options, including associated benefits and risks. While paper-based versions exist, we included only decision aids with computational elements, such as algorithms or structured models, to incorporate patient preferences. These computational decision aids not only provide information but also offer treatment recommendations or personalized insights, bridging the gap between understanding and decision-making. **Decision Support Systems (DSS)** utilize advanced computational techniques to include all relevant information (e.g. patient information, information on the clinical setting, etc. and are most often integrated in clinical information systems) and generate treatment recommendations based on the individual patient data.

### Methods used to incorporate patient preferences

**Classical statistical methods** (n=20; [16, 23, 24, 26, 27, 30, 31, 33–35, 38, 39, 41, 44–46, 49, 51, 58, 61, 63]) often come as plain models and include decision analysis models such as Markov (n=8; [24, 26, 34, 35, 41, 49, 58, 61]) or Bayesian models (n=2; [51, 56]), and prediction models like utility-maximization algorithm (n=3; [16, 27, 28]), as these are used to make probabilistic inferences, and optimize decision-making processes in uncertain environments. **Multi-Criteria Decision Analysis (MCDA)** methods (n=14; [17, 28, 29, 37, 47, 48, 52, 53, 56, 57, 59, 60, 62, 64]) are commonly used for decision aids and are often part of a DSS. They are tailored to facilitate decision-making by considering a wide range of criteria. These methods can balance various factors such as patient preferences, clinical outcomes, costs, and risks, to aid healthcare professionals in making informed decisions. The techniques included within this category, namely the Analytic Hierarchy Process (AHP) (n=2; [48, 62],) Stochastic Multi-criteria Acceptability Analysis (SMAA) (n=2; [52, 60]), and Weighted Sum Model [27]. However, there is a lot of heterogeneity in the methodology for MCDAs. **Decision Trees** (n=7; [22, 23, 25, 30, 32, 43, 55]), serve dual purposes within the realm of medical decision-making. As both a decision-making tool and a statistical model, Decision Trees offer visual and analytical means to predict outcomes and navigate through complex decision processes. Therefore, they are used as plain models, but also as decision aids. Four publications used approaches that do not fit into any of the categories (n=4; [38, 42, 50, 54]). Each uses a unique idea to integrate patient preferences into a medical decision model. This includes customized models, such as artificial intelligence frameworks [29], risk calculators [52], and interactive patient engagement tools, such as decision boxes for clinicians [54]. A glossary for the terms used is in the Appendix A-A1.

### Decision-making frameworks and treatment contexts

The decision-making tools included in this review operate within a variety of treatment contexts, each adapted to address distinct clinical needs and decision-making structures. For instance, tools used in oncology frequently employ Markov models to manage the complexities of neoplasm treatment [9, 14, 49]. Markov models enable clinicians to track disease progression over time, allowing for dynamic adjustments in response to patient changes or new clinical data. This flexibility is crucial in cancer treatment, where sequential treatment choices and evolving patient conditions necessitate continuous updates to align with both clinical goals and patient priorities [37].

In decision contexts that require clear, binary choices-such as opting for surgery versus non-surgical management-decision trees are commonly utilized [15, 24]. These models simplify complex decision pathways into intuitive branches, presenting patients and clinicians with a clear set of options and their respective outcomes. Decision trees are especially effective in cardiovascular decision-making, where patients often face choices between invasive interventions and conservative treatments (e.g., lifestyle changes, medication) [50, 58]. By visually delineating risks and benefits, decision trees support patients in understanding each choice’s implications, helping to guide informed discussions with clinicians [27].

For chronic disease management, multi-criteria decision analysis (MCDA) frameworks and decision aids are particularly advantageous. These tools can accommodate a variety of treatment attributes simultaneously, including potential side effects, ease of adherence, and long-term health benefits [43, 55]. In conditions such as diabetes and hypertension, where patients may prioritize different aspects of care, MCDA provides a structured approach that aligns treatment recommendations with the specific values each patient holds, whether it be minimizing side effects, achieving specific health targets, or simplifying daily treatment routines [56].

Tools tailored to preventive care and risk assessment often employ cost-effectiveness analyses and quality-adjusted life year (QALY) calculations to weigh long-term benefits against potential risks and costs [23, 38, 49]. These methods are especially useful in public health decision-making, such as determining eligibility for preventive screenings or vaccinations. They allow clinicians and policymakers to integrate patient perspectives on life quality and longevity into broader health recommendations, facilitating decisions that are not only evidence-based but also patient-centered [41].

In sum, the frameworks used in these decision-making tools are carefully matched to their treatment contexts, demonstrating the need for adaptable, context-specific approaches. Each framework-whether it be Markov models in oncology, decision trees in cardiovascular care, or MCDA in chronic disease management-plays a critical role in integrating patient priorities within the bounds of clinical goals [53, 57]. This adaptability underscores the importance of choosing the appropriate decision-making framework to ensure that both clinical efficacy and patient values are addressed in each decision.

### Elicitation of patient preferences

In the found literature, two strategies have been identified for eliciting patient preferences. The first is a cohort-based approach (n=14; [17, 27, 34, 36, 40, 42, 47, 49, 51, 52, 54, 60, 62, 64]), which utilizes patient preferences gathered from various sources such as literature reviews (n=6; [27, 36, 51, 52, 57, 64]), focus groups (n=1; [45]), or Delphi methods (n=2; [40, 54]). These sources help to define a set of preferences that are relevant to patients. These predefined preferences are then presented to individual patients, who assign personal weights to them, to yield individualized recommendations. The second strategy is a more personalized approach, where tools are employed to elicit patient preferences individually. This method ensures consistency and reliability in data collection through standardized techniques. Key methods used in this approach include Discrete Choice Experiments (DCEs) (n=2; [39, 45]), where patients choose between different sets of treatment options to identify the most important attributes in their decision-making process. Conjoint Analysis (CA) [53] is a survey-based statistical method that quantifies how patients value different treatment attributes by presenting them with hypothetical scenarios. Standard Gamble (SG) (n=5; [22, 24, 28, 33, 35, 58]) is a method used to measure preferences under conditions of uncertainty. Patients are asked to choose between a certain outcome and a gamble with possible better or worse outcomes, helping to quantify their risk tolerance. The Analytic Hierarchy Process (AHP) (n=2; [48, 62]) is a structured method that helps patients prioritize and make the best decisions by quantifying their preferences. Time Trade-Off (TTO) [22] is a method where patients are asked to trade off time in their current health state against time in a more desirable health state, thereby quantifying their preferences for different health outcomes. The literature also highlights various specific methods and tools designed to assess patient preferences, further enhancing the capability to provide personalized treatment recommendations. [38, 41, 42, 53]

One study uses a threshold utility analysis [22], to determine the utility values at which patient preferences for different health states change. Measures like quality-adjusted life expectancies (QALEs) and quality-adjusted life years (QALYs) (n=3; [23, 27, 49]) quantify both the quality and quantity of life lived, integrating patient preferences for different health states. Decision analysis models, including Markov decision analysis (n=2; [49, 52]) and Monte Carlo simulations [37], play a role in this process. Preference elicitation techniques, such as the swing weighting method [55], are essential to capture patient risk preferences and criteria weights. Multi-attribute utility models (MAUM) [41] are used to evaluate multiple treatment attributes based on patient preferences. Graphical rating scales [34] and methods such as direct rating and best-worst scaling [38] provide straightforward ways to quantify patient preferences. Discrete choice experiments (n=2; [35, 60]) determine how patients value different treatment attributes by presenting them with a series of choices. Normalization and sensitivity analysis are used to make sure patient preferences are applied consistently and to test how changes in preferences affect outcomes. Classical statistical methods (n=2; [44, 58]) analyze and quantify patient preferences. Survey-based quantification (n=3; [33, 43, 54]) and qualitative to quantitative conversion [53] methods convert patient-expressed preferences into quantitative scales. Other methods, such as independent percent contribution [37] and re-weighting [43], also contribute to the balance of patient preferences.

### What patient preferences have been incorporated

Although some studies highlighting a holistic and open approach to patient involvement and a general unrestricted approach to involve patient preferences (n=5; [29, 35, 46, 59, 61]), most of the evidence provides a list of specific patient preferences that are incorporated (n=40; [16, 17, 22–34, 36–45, 47, 48, 50–58, 60, 62–64]). Our analysis can be categorized into several key themes. A significant number of studies (n=12; [16, 24, 28, 33, 37, 42, 44, 52, 56, 62, 64]) integrate patient preferences related to specific health outcomes and risks. These preferences encompass individual risks such as stroke, major bleeding, and intracerebral hemorrhage, as well as concerns about potential side effects of treatments. Preferences regarding medication types and treatment attributes are frequently considered (n=5; [37, 38, 43, 44, 58]). This includes choices between oral versus injectable medications, monitoring schedules, meal times, self-monitoring, and attributes such as frequency, associated risks, and costs. Several studies focus on the utilities and outcomes of different treatment regimens (n=2; [23, 57]), emphasizing the subjective value patients place on various health states. Preferences related to resuscitation and end-of-life care [27] are also incorporated, ensuring that models respect patient wishes regarding life-sustaining treatments and end-of-life decisions. Many studies consider preferences that impact quality of life and functional outcomes (n=4; [24, 32, 39, 62]). This includes the ability to walk, ease of prosthesis use, pain levels, healing after surgery, and the ability to return to work or other activities.

### Involvement of End Users

The majority of these methods are designed to cater to both patients and clinicians, (n=28; [22, 24, 25, 27–29, 31, 32, 34–36, 40, 43, 45, 48–51, 53–55, 57, 59, 60, 62, 64]). In contrast, a smaller subset of methods is solely targeted towards patients (n=9; [16, 23, 26, 39, 41, 46, 47, 58, 61]), focusing primarily on empowering individuals with the information and tools needed to make informed healthcare decisions independently. In addition, there are methods that also incorporate the interests of healthcare providers (n=8; [17, 30, 33, 37, 42, 52, 56, 63]), emphasizing organizational priorities, efficiency, and resource management within healthcare settings. Each of these approaches reflects different priorities and emphasizes the importance of considering diverse perspectives in medical decision support systems.

### End user acceptance

End user acceptance of computational healthcare decision-making methods for incorporating patient preferences, is crucial from both the patients’ and clinicians’ perspectives. Our investigation shows that the acceptance of patients and clinicians has not been the focus of most publications (n=24; [16, 22, 23, 25–28, 30, 31, 34, 36, 37, 39, 40, 42, 46, 48, 51, 52, 55, 56, 60, 63, 64]). When end user acceptance was tested or was planned to be tested (n=21) the majority focused on the patient’s perspective (n=20; [17, 24, 29, 32, 33, 35, 37, 38, 41, 43–45, 47, 49, 50, 53, 55, 57, 61, 62]). Some publications performed an alpha/pilot test (n=4; [17, 45, 50, 62]) or even a beta test [17]. Most of the tools had a positive outcome in terms of acceptance by the end users (n = 16). However, patients have not accepted only one tool [23]. In addition, clinicians tested only one tool, with a negative result [24].

### Sources of heterogeneity in decision-making tools

To clarify the sources of heterogeneity observed in the reviewed decision-making tools, we have categorized the main characteristics contributing to variability in patient preference integration (see Table 2). The table outlines five key characteristics: the type of decision support, use case, methods for incorporating preferences, elicitation techniques, and end user involvement. Each characteristic contributes uniquely to the observed differences. For instance, the type of decision support tool - whether a statistical model, decision aid, or decision support system - influences complexity and transparency. Similarly, the method of preference elicitation, such as discrete choice experiments or time trade-offs, impacts how preferences are quantified and integrated. By categorizing these sources of heterogeneity, Table 2 provides a structured view of the diverse approaches to incorporating patient preferences in clinical decision-making, highlighting areas for potential standardization.

**Table 2:** Sources of heterogeneity in decision-making tools and patient preference integration

Characteristic	Categories	Heterogeneity
**Type of Decision Support**	Model, Decision Aid, DSS	Different types of decision support vary in complexity and transparency. Models often use statistical or algorithmic approaches, while Decision Aids and DSS are more likely to involve interactive tools or systems integrated with clinical information [14, 15].
**Use Cases**	Neoplasms, Circulatory Diseases, Nervous System, Others	Focus varies by disease, with most applications in complex, high-incidence diseases (e.g., cancers, cardiovascular). Disease type often influences the depth of patient preference integration, as more complex diseases benefit more from patient input [9].
**Method of Incorporating Preferences**	Statistical Models, MCDA, Decision Trees	Statistical models provide probabilistic outcomes, MCDA balances multiple criteria, and Decision Trees offer visual decision pathways. MCDA is commonly part of DSS, while Decision Trees can function as both models and aids, adding methodological diversity [43, 54].
**Elicitation of Patient Preferences**	Standard Gamble, Time Trade-Off, DCE, Conjoint Analysis	Different elicitation methods impact how preferences are quantified; for example, DCE and Conjoint Analysis emphasize attribute importance, while Standard Gamble and Time Trade-Off measure risk tolerance and preferences under uncertainty [55].
**Involvement of End Users**	Patient, Clinician, Both	Tools designed for patient-only use emphasize empowerment, while those for both patients and clinicians aim to facilitate shared decision-making. Clinician-targeted tools often incorporate organizational priorities and resource management considerations [35, 37].

### Limitations discussed by authors

The authors of the studies included in this review recurrently named several limitations that can affect the generalizability and applicability of their findings. Many studies had small sample sizes or limited participant diversity, restricting the ability to generalize the results to broader populations (n= 5; [22, 39, 61, 62] ). Issues with cost reporting were also prevalent, with studies often reporting charges instead of true costs. This discrepancy affects the accuracy of economic evaluations and was noted in multiple studies involving economic assessments (n=2; [30, 49]). Participation and engagement were significant concerns, as many studies faced low participation rates, with a substantial number of patients either unwilling or unable to participate. This low engagement impacts the validity of the results and suggests that such decision aids may not be suitable for all groups of patients [23]. Decision-analytic models frequently did not consider all relevant factors or treatments and were often based on outdated data. These models had limitations specific to their context, restricting the applicability of the results and underscoring the need for updated and comprehensive models (n=4; [24, 34, 40, 64]). Technological and accessibility barriers were also identified. The applicability of internet-based decision aids was limited in populations lacking internet access. Furthermore, some tools had technical limitations or were not user-friendly, affecting their generalizability to other medical decisions and contexts (n=2; [17, 41]). Several studies lacked detail on their measurement scales or did not capture the full spectrum of patient experiences, raising concerns about the comprehensiveness and feasibility of their assessments. This issue was particularly evident in studies with limited health state considerations or detailed feasibility evaluations (n=3; [16, 50, 63]). Context-specific issues were another common theme. Studies conducted in specific settings, such as Veterans Affairs medical centers, or those that did not incorporate costs, had limited applicability to other settings or broader populations. This limitation highlights the need for contextually adaptable frameworks and broader applicability of study findings (n=2; [48, 49]). A summary of the key findings of each section is provided in Table 3.

**Table 3:** Summary of the key findings of each section

Section	Description	Key Findings
Definitions of Patient Preferences	Provides an overview on the various definitions of patient preferences that the publications contain.	Utility-based definitions offer numerical clarity but risk oversimplification. Trade-off focused definitions highlight balancing risks and benefits but need more nuanced models. Elicitation-based definitions systematically capture priorities but lack adaptability. Context-specific definitions address variability across conditions, while value alignment definitions emphasize aligning decisions with broader patient goals.
Use Cases	Summarizes the specific applications and medical conditions (e.g., oncology, cardiology) where patient preferences are incorporated.	Oncology and cardiology are the most common use cases, with applications focused on high-incidence, complex conditions where patient values are highly relevant. Chronic disease management tools prioritize patient preference to manage treatment adherence and quality-of-life aspects, especially in diseases like multiple sclerosis and diabetes.
Type of Decision Support	Identifies types of decision support tools such as models, decision aids, and decision support systems (DSS) employed.	Decision aids and DSS tools are widely used to facilitate shared decision-making; they help both patients and clinicians navigate complex decisions with high patient involvement. Models aiding in more accurate and personalized medical planning.
Methods Used	Outlines statistical and MCDA methods as well as specific models like Markov models, Bayesian networks, etc.	MCDA is frequently applied to balance clinical outcomes with individual preferences, especially in chronic diseases. Statistical models like Markov chains for tracking disease progression and Bayesian networks for probabilistic risk modeling are commonly used to integrate patient preferences, improving alignment with personalized treatment goals.
Frameworks & Treatment Contexts	Describes how decision-making tools are matched to different clinical contexts (e.g., chronic disease, preventive care) with framework examples.	In oncology, Markov models accommodate sequential treatment needs, adjusting for changing patient conditions and preferences over time. Preventive care uses frameworks like cost-effectiveness analysis (CEA) and QALY-based models to weigh patient quality of life and longevity in health recommendations, supporting screenings and lifestyle interventions.
Elicitation of Patient Preferences	Details various techniques (e.g., Standard Gamble, Conjoint Analysis, DCEs) used to capture individual patient preferences.	Standard Gamble quantifies patient risk tolerance, while Conjoint Analysis and Discrete Choice Experiments (DCE) capture relative importance of treatment attributes, allowing personalized risk-benefit profiles. Analytic Hierarchy Process (AHP) and Time Trade-Off (TTO) are used for prioritizing long-term health states and outcomes.
Patient Preferences Incorporated	Lists the specific patient preferences, including risk tolerance, treatment attributes, and outcome priorities integrated into models.	Patient preferences span risk tolerance, treatment invasiveness, side effect concerns, and quality of life. Preferences vary by condition, with cancer patients often valuing survival outcomes, while chronic disease patients may prioritize treatment simplicity and minimizing side effects to improve adherence.
End User Involvement	Identifies the primary end users targeted by these tools, such as patients, clinicians, or both, and their roles in decision-making.	Most tools aim to enhance shared decision-making between patients and clinicians, with features designed to address clinician concerns around time efficiency and usability. Some tools target patients exclusively, focusing on empowerment through easy-to-understand visuals and interactive options for preference adjustments.
End User Acceptance	Summarizes levels of acceptance of these tools by patients and clinicians, highlighting any pilot or beta testing outcomes.	Studies show high acceptance rates among patients in pilot testing, where tools often lead to greater satisfaction and engagement in decision-making. Clinician acceptance, however, requires additional support, as barriers like workflow integration and complexity persist, calling for tailored training programs.
Sources of Heterogeneity	Explains the main sources of variability in preference integration, such as differing model types, elicitation methods, and user involvement.	Heterogeneity arises from variations in decision-making models, ranging from basic statistical tools to complex MCDA and utility-based frameworks. Differences in preference elicitation methods, such as DCE versus Standard Gamble, and the varying roles of patients and clinicians further add complexity.

## Discussion

### Definition of patient preferences

This scoping review identifies five key categories of patient preference definitions. These categories reflect the evolving understanding of preferences, emphasizing personalization in healthcare. Utility-based definitions provide quantitative clarity but may oversimplify multidimensional preferences. Trade-off focused definitions highlight balancing competing risks and benefits but require more nuanced models to address variability. Elicitation-based definitions systematically capture patient priorities but may lack adaptability in complex settings. Context-specific definitions underscore the variability of preferences across clinical scenarios, while value alignment definitions stress aligning healthcare decisions with patients’ broader goals and priorities. The results reveal strengths in categorizing definitions but also highlight challenges in their consistent application. For example, integrating context-specific definitions with value alignment principles could enhance shared decision-making frameworks. Similarly, more adaptable elicitation methods could better accommodate dynamic or nuanced preferences.

### Patterns in clinical applications and focus on specific conditions

Of the 45 publication, predominantly in neoplasms (e.g., prostate, breast, and ovarian cancers) and circulatory diseases (e.g., atrial fibrillation, ischemia). The focus on these areas likely reflects both the high incidence of these diseases and the complexity of treatment decisions, which often benefit from patient-centered approaches [15]. Integrating patient preferences in complex disease management aligns well with the goals of patient-centered care, allowing treatment options to reflect individual values and preferences. This finding suggests that patient preference integration may be particularly valuable in diseases with complex treatment paths, where patients’ values and perspectives play a central role in decision-making [22].

### Methodological trends and the evolution of patient preference models

Our review highlights an evolution in methodological approaches. Initially, simpler statistical models dominated the field, but since 2010, Multi-Criteria Decision Analysis (MCDA) and Decision Support Systems (DSS) have become more prevalent [54]. These tools have gained traction as they offer structured, transparent approaches to balancing multiple factors, which is critical in patient-centered treatment. MCDA-based methods, especially in decision aids, balance clinical outcomes with individual patient preferences, reinforcing the growing trend toward patient-inclusive, personalized models. This shift mirrors the broader move toward shared decision-making and the increased digitalization in healthcare [43], demonstrating an increasing demand for models that align treatment options with patients’ unique values and needs.

### Challenges of heterogeneity and standardization

The substantial heterogeneity in methods for incorporating patient preferences reveals both the adaptability of these models and a need for standardization across studies. While flexibility allows for customization to specific patient needs and clinical contexts, the inconsistency in methods can limit their comparability and generalizability [55]. Developing adaptable yet standardized frameworks could improve reliability without sacrificing clinical relevance. Standardization efforts might involve creating guidelines or adaptable frameworks (e.g. EVIDEM and TRIPOD) that ensure consistent application of core principles while allowing modifications based on context.

### Impact on patient outcomes and need for clinician acceptance

Incorporating patient preferences has been shown to influence treatment choices significantly, with studies reporting substantial changes in recommendations when preferences are considered [23, 24]. Improved patient satisfaction, adherence, and health outcomes highlight the potential benefits of patient-centered decision models [22, 33]. However, achieving clinician acceptance of these models poses challenges. Clinicians may hesitate due to concerns over time constraints, perceived complexity, and insufficient training [35, 37]. Addressing these barriers requires integrating user-friendly tools into clinical workflows and enhancing clinician education on incorporating patient preferences effectively. For example, user-centered design and early-stage clinical validation could make these tools more accessible and aligned with workflow requirements [56]. Demonstrating the models’ benefits in enhancing shared decision-making, patient satisfaction, and clinical outcomes could further foster clinician engagement. However, this does not address the cultural challenges, as clinicians are often the primary decision-makers, reinforcing the need to ensure that patient-centered models genuinely shift the balance toward shared decision-making rather than reinforcing clinician authority.

### Gaps in end user engagement and future research directions

Despite the importance of clinician and patient engagement in these tools, few studies have directly explored these aspects. Our findings indicate a need for more research on clinician acceptance, as end-user engagement is crucial for the models’ effectiveness [34, 49]. Future studies should emphasize co-designing tools with clinicians to ensure alignment with practical needs, pilot implementations to demonstrate real-world value, and assessments of the impact on shared decision-making, patient satisfaction, and adherence [31, 50]. Developing frameworks that promote engagement from both patients and clinicians could ensure these tools’ successful integration into clinical practice, facilitating broader adoption and greater impact.

### Implications for future research mentioned by the authors

This scoping review identified several key areas for future research to improve decision-making tools and their applicability in clinical settings. A prominent topic is the need for larger and more diverse sample sizes to validate findings and enhance generalizability (n=2; [22, 39]). In addition, prospective and longitudinal studies are needed to track patients over extended periods, prioritize patient-centered outcomes, and utilize advanced analytical methods such as simulation modeling and machine learning [30]. Evaluation and improvement of decision-making tools are crucial. Future research should focus on testing utility assessment software that requires minimal input from researchers to eliminate bias effects [23]. Furthermore, it is essential to evaluate and improve decision-analytic models by incorporating recent data and exploring the best methods for utility assessment [55]. Optimizing the timing and interface of decision aids, as well as conducting efficacy studies to assess whether tools work in ideal circumstances and investigating discrepancies between patient decisions and model outcomes, are also important areas (n=2; [28, 35]). Validation of findings in diverse patient populations and clinical settings was identified as another critical area. This includes incorporating additional clinical factors, performing user acceptance tests and evaluating the long-term outcomes of treatment decisions [56]. Furthermore, exploration of the implementation and scalability of decision aid tools in clinical settings and their generalizability to other contexts was considered important [48]. Effectiveness and impact studies are necessary to ensure the utility of decision-making tools. The conduct of randomized controlled trials (RCTs) and efficacy studies to explore generalizability, evaluate usability with low health literacy, and incorporate patient feedback are essential steps (n=3; [17, 32, 61]). Personalization and customization of tools should be enhanced to better cater to individual patient needs and preferences. Further testing of the potential effects on quality of life and functioning, as well as evaluations from healthcare professionals, should be conducted. Simplifying tools with enhanced personalization will maximize the benefits of evidence- and preference-based approaches [47]. Addressing methodological challenges is also critical. Larger roll-outs, tool modifications based on pilot testing results, addressing challenges such as blinding, and implementing uncertainty analysis are necessary steps to improve decision-making tools [62]. Conducting comparative effectiveness research to compare outcomes based on individual patient preferences with traditional approaches will further enhance the utility of these tools [34].

### Limitations and recommendations for future research

This review highlights several areas for future exploration, such as the need for larger, diverse sample sizes, improved cost-reporting accuracy, and broader clinical scenarios to enhance generalizability [55]. Future studies should focus on advancing personalization and addressing methodological challenges through robust validation in diverse settings. Specific research directions could include assessing clinician and patient engagement across healthcare settings and exploring how these tools perform across health systems. By broadening the application and effectiveness of patient-centered decision models, future research can contribute to more responsive, individualized healthcare [14]. At last, future research should prioritize establishing and incorporating guidelines as EVIDEM or TRIPOD to enhance consistency across diverse clinical environments.

### Strength and limitations of this study

To our knowledge, our scoping review provides the first comprehensive overview of practical approaches of computational methods that integrate patient preferences at an individual level to deliver personalized treatment recommendations. Unlike previous literature, which often addresses patient preferences in broader contexts this review focuses specifically on computational approaches and real-world applications of integrating patient preferences within clinical settings. By mapping current methods and models, this review was able to show gaps and trends in the practical use of medical decision model that incorporate patient preferences. It is based on a peer-reviewed scoping review protocol using the JBI methodology and encompasses a broad search, including both white and gray literature. This extensive search includes reviews, conference abstracts, dissertations, theses, technical reports, relevant documents from reputable sources, clinical practice guidelines, reports, policy documents, online resources, and expert opinions obtained through personal communication. The review demonstrates the wide variety of existing applications, from common to unusual, showcases different types of decision-making support, and categorizes these methods by their underlying methodologies. The longitudinal analysis allows for an examination of the topic’s popularity and the identification of emerging trends. Additionally, the review provides an overview and description of methods to quantify, balance, and evaluate patient preferences within these approaches. By summarizing described limitations and future implications, it identifies common issues and research gaps. However, the study has notable limitations. The findings on the evaluation of patient preferences, as well as end user acceptance, have limited validity since no follow-up studies were included. One key limitation of this review is the potential for publication bias. Studies with positive or significant findings are more likely to be published, while those with negative or inconclusive results may be underrepresented. This could skew the evidence base toward more favorable reports of methods incorporating patient preferences into medical decision models, limiting the generalizability and robustness of our conclusions. Additionally, a few studies (n=5) could not be accessed, which may further limit the comprehensiveness of the review. Lastly, the analysis includes only practical applications. We intend to conduct a second scoping review that will specifically focus only on methodological methods in the future.

## Conclusion

This scoping review highlights the considerable diversity of practical approaches for computational methods used to integrate patient preferences into medical decision models. While this variability allows tailoring to specific clinical contexts, it also underscores a notable absence of the usage standardized frameworks that could enhance consistency and comparability across applications. However, these frameworks should acknowledge individual values, priorities, and trade-offs that individuals place on healthcare outcomes, interventions, and processes, reflecting their unique circumstances, goals, and broader life context. The incorporation of patient preferences has shown significant influence on treatment decisions, particularly in fields such as oncology and cardiology, where aligning care with patient values is essential.

There is an emerging trend towards the integration of more and diverse methods to support patient-centered care. This trend, however, points to a critical gap in the development of cohesive frameworks that can balance flexibility with reproducibility. Addressing this gap will be crucial for fostering clinician acceptance and applicability across various healthcare settings, as standardized, adaptable methodologies may better support the complexity of shared decision-making.

An additional challenge lies in the limited focus on end-user engagement, particularly with clinicians and patients who are essential for the successful implementation and impact of these tools. Our findings suggest a pressing need for more research on clinician acceptance and engagement, as real-world utility relies on the alignment of these models with clinical practice requirements. Future studies should prioritize co-designing decision-making tools with clinicians to ensure practical alignment, piloting these tools to validate real-world applicability, and evaluating their effects on shared decision-making, patient satisfaction, and adherence.

In summary, advancing these tools requires not only methodological refinement but also frameworks that actively engage both patients and clinicians. Developing robust, reproducible methodologies that integrate patient preferences can transform shared decision-making and improve patient-centered care, facilitating broader adoption and lasting impact in clinical settings.

## Supplementary Information

Supplementary Material 1: Appendix A.

Supplementary Material 2: Appendix B.

Supplementary Material 3: Appendix C.

Supplementary Material 4: Appendix D.

## Data Availability

Relevant data are provided within the manuscript or in the supplementary information files. The raw datasets used and/or analyzed during the current study are available at https://github.com/Fusiakja/EPAMeD.
